# Mouse models for muscular dystrophies: an overview

**DOI:** 10.1242/dmm.043562

**Published:** 2020-02-21

**Authors:** Maaike van Putten, Erin M. Lloyd, Jessica C. de Greef, Vered Raz, Raffaella Willmann, Miranda D. Grounds

**Affiliations:** 1 Leiden University Medical Center, Department of Human Genetics, Leiden, 2333 ZA, The Netherlands; 2 The University of Western Australia, School of Human Sciences, Perth 6009, Australia; 3 University of Basel, Biozentrum, Basel, CH-4056, Switzerland

**Keywords:** Disease pathology, Mouse models, Muscular dystrophy

## Abstract

Muscular dystrophies (MDs) encompass a wide variety of inherited disorders that are characterized by loss of muscle tissue associated with a progressive reduction in muscle function. With a cure lacking for MDs, preclinical developments of therapeutic approaches depend on well-characterized animal models that recapitulate the specific pathology in patients. The mouse is the most widely and extensively used model for MDs, and it has played a key role in our understanding of the molecular mechanisms underlying MD pathogenesis. This has enabled the development of therapeutic strategies. Owing to advancements in genetic engineering, a wide variety of mouse models are available for the majority of MDs. Here, we summarize the characteristics of the most commonly used mouse models for a subset of highly studied MDs, collated into a table. Together with references to key publications describing these models, this brief but detailed overview would be useful for those interested in, or working with, mouse models of MD.

## Abstract (precis)

**Summary:** This Review and its accompanying comprehensive table summarize the most commonly used mouse models for a subset of highly studied muscular dystrophies.

## Introduction

Muscular dystrophies (MDs) are a clinically and genetically heterogeneous group of inherited disorders. They are characterized by progressive muscle weakness affecting skeletal muscles, but some MDs involve cardiac and/or smooth muscles (Emery, 2002; Mercuri and Muntoni, 2013). Age of onset, disease severity and progression varies markedly between the different MDs. To date, more than 50 causative genes have been identified. Historically, MDs were classified based on the main clinical manifestations and the age of onset. Later, the mode of inheritance was also taken into account, resulting in further sub-classification of limb-girdle muscular dystrophy (LGMD) and congenital muscular dystrophy (CMD).

## Importance of mouse models for studying disease mechanism and potential therapies

The availability of animal models of MDs plays a key role in studying disease pathology. Despite differences in some pathological hallmarks compared to humans, animal models have provided important insights into causal gene relationships and into the functional cellular and molecular mechanisms of disease pathogenesis. Consequently, a variety of therapeutic approaches have been developed using these models for MDs. Animal models play a pivotal role in preclinical studies to progress therapies to the clinic, from proof-of-principle studies, dosage and efficacy studies to extended preclinical trials (Allamand, 2000; Durbeej and Campbell, 2002).

Mice are the most frequently used models of MDs, as they are easy and relatively inexpensive to breed and maintain in large numbers, and to handle, treat and genetically modify. They are ideal subjects for preclinical studies owing to their small body size, short gestation and life span, and the abundance of experimental reagents available, such as antibodies and expression constructs. In addition, the mouse genome is well characterized and is largely comparable to the human genome. Moreover, detailed natural life-history data are available for an increasing number of mouse strains, providing crucial information for the accurate design of preclinical studies. Especially in the last decade, the research community has highlighted the need for detailed natural life-history data from both MD patients and the mouse models. This call arose due to the failure of several drugs in clinical trials despite encouraging preclinical data (Kornegay et al., 2014; Straub and Mercuri, 2018). As such, multiple international initiatives aim to improve preclinical trial design and execution (Gordish-Dressman et al., 2018; Heslop et al., 2015; Nagaraju et al., 2009). The TREAT-NMD Alliance has coordinated the generation and maintenance of standard operating procedures (SOPs) for several widely used outcome measures for the most commonly used mouse models of Duchenne muscular dystrophy (DMD) (Nagaraju et al., 2009; Willmann et al., 2011a), spinal muscular atrophy (SMA) (Willmann et al., 2011b) and CMD (Saunier et al., 2016). Detailed information is available on the TREAT-NMD website (https://treat-nmd.org/research-overview/preclinical-research/). These SOPs have now been downloaded worldwide more than 11,000 times in the last 7 years, and have been implemented in many research publications (Carlson et al., 2011; Mantuano et al., 2018; Mele et al., 2019; Tam et al., 2015; Zschüntzsch et al., 2016). It is hoped that implementation of the SOPs reduces intra- and inter-variability between complying laboratories. For mouse models of other MDs, these initiatives are either ongoing or planned.

In Table 1, we provide a detailed overview of the main disease characteristics of the most commonly used mouse models in preclinical research for nine MDs, with a focus on those used extensively in preclinical trials and those that were crucial to elucidate aspects of the pathology of each MD.

**Table 1.**
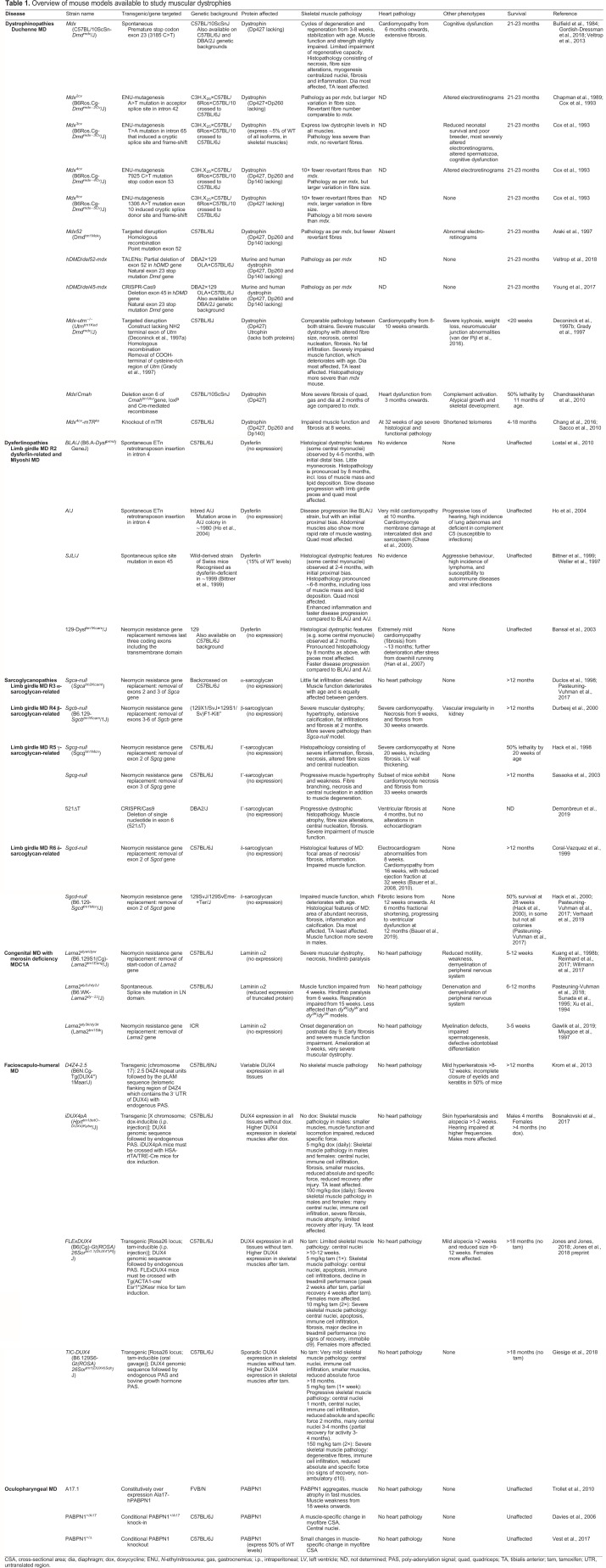
Overview of mouse models available to study muscular dystrophies

## Technologies to generate mouse models for MDs

Naturally occurring dystrophic mouse strains, in which a spontaneous mutation results in an MD phenotype (e.g. *mdx*, A/J, SJL/J and *dy^2J^/dy^2J^*), make up the minority of the available models. The majority of the mouse models have been genetically engineered, either by overexpressing the mutated gene or replacing the wild-type (WT) gene using a variety of non-targeted or targeted methods. Here, we briefly explain each of these approaches. For more comprehensive reviews on gene-editing techniques for the generation of mouse models, we refer the reader to Gurumurthy and Lloyd (2019), Hall et al. (2009) and Justice et al. (2011).

### Non-targeted gene disruption

Some strains described in Table 1 have been generated using a non-targeted approach, by chemicals that randomly induce point mutations throughout the genome. *N*-ethylnitrosourea (ENU) is a commonly used mutagen that randomly mutates the DNA with a frequency of 1 mutation per 700 loci (Stottmann and Beier, 2014). The offspring of ENU-exposed mice are then screened for a marker for the disease: for instance, in the case of *mdx^2–5cv^* models, female offspring were screened for muscle dystrophy by assessing creatine kinase (CK) levels in blood, a marker of muscle leakiness (Chapman et al., 1989; Cox et al., 1993; Im et al., 1996). Carrier mice with elevated CK levels were then further investigated for muscle pathology, and their genomes were subsequently sequenced to identify the specific mutation.

### Targeted gene disruption

The majority of the knockout mouse models that are available for MDs were generated via gene targeting. There are several protocols, but they all employ the cell-intrinsic homologous recombination DNA repair mechanism to insert a targeting vector in a homologous genetic locus of interest (Hall et al., 2009). Consequently, cells lack the targeted sequences (i.e. one or multiple exons) and fail to express the corresponding protein. The procedure requires a vector, which has a specific make-up depending on the method used. Generally, this vector contains the sequences of the regions flanking the exon(s) of interest and, in the middle of this, a drug selection marker (like that for neomycin resistance), which replaces the exon(s) of interest in the cell and allows for cell selection (Bouabe and Okkenhaug, 2013). Some vectors also contain recombinase-binding elements such as LoxP or Flp recombination target sites. Via electroporation, the vector is introduced into murine embryonic stem cells, which are then cultured and selected based on the resistance for the selection marker of choice. The resistant cells are then injected into a mouse blastocyst from which a knockout offspring develops.

The transcription activator-like effector nuclease (TALEN) technology (Cermak et al., 2011) has been used to generate the hDMD/del52-*mdx* model (Veltrop et al., 2018). For this model, TALENs induced double-strand breaks in the region of interest of a gene. Consequently, cells repaired these breaks through the non-homologous end-joining (NHEJ) DNA repair process. This is the dedicated repair mechanism to restore double-strand DNA breaks in non-dividing cells, in which DNA ends are ligated without the use of a template in an error-prone manner that typically disrupts the open reading frame, knocking out the gene of interest.

Recently, CRISPR/Cas9 technology has been used to rapidly engineer precise human mutations, generating many new mouse models (Cong et al., 2013). Several variations of the CRISPR/Cas9 system have been used, e.g. to generate the hDMD/del45-*mdx* (Young et al., 2017) and *Dmd*del8-34 models for DMD (Egorova et al., 2019), and the 521ΔT model for LGMD R5 γ-sarcoglycan-related (Demonbreun et al., 2019). For the DMD models, guide RNAs were designed to target the region of interest and to guide the Cas9 nuclease to this region to execute the cuts. NHEJ ensured the deletion of this particular region. The 521ΔT model for LGMD R5 γ-sarcoglycan-related (Demonbreun et al., 2019), on the other hand, is a knock-in model: here, a mutated part of exon 6 was used as repair template, which replaced the intact intrinsic exon 6 sequence upon a CRISPR/Cas9-mediated DNA break and homology-directed repair.

## Dystrophinopathies

DMD is an X-linked progressive disorder caused by mutations in the *DMD* gene that result in the lack or defective forms of the structural muscle protein dystrophin and manifests in young children. The most commonly used DMD model is the *mdx* mouse (Bulfield et al., 1984; Willmann et al., 2009) and, to a lesser extent, its genetic variants *mdx*^2cv^, *mdx^3cv^*, *mdx^4cv^*, *mdx^5cv^* (Cox et al., 1993), *mdx*52 (Araki et al., 1997) and DMD^null^ (Kudoh et al., 2005). *Mdx* mice are primarily affected from 3 weeks onwards, when cycles of muscle necrosis and regeneration occur during the intense growth period. These cycles continue until ∼12 weeks of age, when 80% of myofibres have central myonuclei indicating past events of necrosis and regeneration (Coulton et al., 1988). Thereafter, the pathology stabilizes and adult mice have greatly reduced incidence of myonecrosis and mildly increasing fibrosis (Grounds, 2008). The diaphragm is more severely affected than other skeletal muscles because of impaired regeneration. Unlike in DMD patients, fat infiltrates are rarely seen in *mdx* mice. Cardiomyopathy is observed in *mdx* mice from ∼6 months of age.

To allow the use of human-specific sequences when investigating the potential of gene therapies (Verhaart and Aartsma-Rus, 2019; Aartsma-Rus and van Putten, 2020), mice carrying mutations in the human *DMD* gene have been generated [e.g. with a deletion of exon 45 in hDMD/del45-*mdx* (Young et al., 2017) or exon 52 in hDMD/del52-*mdx* strains (Veltrop et al., 2018)]. Natural life-history data are not yet available for these new humanized mouse strains, but their pathology appears to be similar to that of the classic *mdx* mouse (Veltrop et al., 2018; Young et al., 2017).

The *mdx* model is limited by its mild disease presentation and only slightly reduced lifespan. To overcome this, several additional mutations were crossed onto the *mdx* background generating double knockout mice. The *mdx-utrn^−/−^* mouse, which lacks dystrophin and its homologue utrophin, is most widely used (Deconinck et al., 1998; Grady et al., 1997). As utrophin is important for neuromuscular transmission, this double knockout is more severely affected and dies before ∼13 weeks of age owing to muscle weakness and respiratory problems. The *mdx-utrn^−/−^* mice also develop kyphosis and heart pathology at <8 weeks of age. These mice are therefore more useful to study survival. However, when evaluating drugs targeting pathology in this model, it is unknown whether a potential improvement is due to addressing pathology induced by lack of dystrophin or that induced by lack of utrophin; thus, it is difficult to determine their translational relevance to DMD.

Two newer double knockouts are the *mdx*/Cmah^−/−^ (Chandrasekharan et al., 2010) and *mdx^4cv^/mTR^ko^* strains (Sacco et al., 2010). Unlike mice, humans carry an inactivating deletion in the cytidine monophospho-*N*-acetylneuraminic acid hydroxylase (*CMAH*; also known as *CMAHP*) gene, which prevents glycosylation with *N*-glycolylneurominic acid. The *mdx*/Cmah^−/−^ model also has a ‘humanized’ mutation in the *Cmah* gene and exhibits a more severe pathology (Chandrasekharan et al., 2010). This double knockout mouse also has impaired life span, with a 50% survival rate at 11 months of age. They have abundant fibrosis in skeletal muscles from 6 weeks onwards, and impaired heart function at 3 months (Betts et al., 2019). Growth and skeletal development is, however, atypical and does not reflect the human DMD trajectory (Wood et al., 2020).

The *mdx^4cv^/mTR^ko^* mouse lacks the RNA component of telomerase and consequently has shortened telomeres, which are closer to the size observed in humans (Sacco et al., 2010). Telomeres protect chromosome ends from deterioration, and their length dictates the replicative lifespan of cells. It was hypothesized that the excellent regenerative capacity of *mdx* mice partly results from long telomeres in mice, and thus shortening the telomeres would impair muscle regeneration. The regenerative capacity of *mdx^4cv^/mTR^ko^* mice is indeed impaired. Skeletal muscle function is affected at 8 weeks, with severe cardiac dysfunction observed in 32-week-old *mdx^4cv^/mTR*^G2^ mice (Mourkioti et al., 2013). As telomere length shortens with each generation of *mdx^4cv^/mTR* mice, the lifespan of second-generation mice is shorter than that of the first generation (Sacco et al., 2010). Nonetheless, the *mdx^4cv^/mTR^ko^* strain has not yet been widely used in the research community.

Another way to exacerbate the disease pathology of *mdx* mice is to cross them onto a different genetic background (McGreevy et al., 2015). For example, the dystropathology worsens when *mdx* mice are bred on the DBA2/J background. The muscle function of this strain, called D2-*mdx*, is severely affected; their muscles are atrophic with extensive fibrosis and initial calcification that largely disappears with age (Coley et al., 2016; Gordish-Dressman et al., 2018; van Putten et al., 2019). Lastly, there are also several immune-deficient *mdx* strains, and *mdx* strains with mutations in additional genes as described in McGreevy et al. (2015). Owing to space restrictions, we did not include these in Table 1.

## Dysferlinopathies

Dysferlinopathies are caused by lack of functional dysferlin, a membrane-associated calcium-binding protein involved in membrane repair. The pathologies usually manifest in young adults as Myoshi myopathy or LGMD R2 dysferlin-related (previously known as LGMD2B; Straub et al., 2018). In general, dysferlin-deficient (dysf^−/−^) mice mimic human dysferlinopathies, show a comparable disease progression with late-onset and similar, though milder, histopathological features, including loss of muscle mass, lipid droplets within slow twitch myofibres, adipocyte replacement of myofibres and inflammation (Grounds et al., 2014; Hornsey et al., 2013; https://www.jain-foundation.org/scientific-resources/research-tools/mouse-models-dysferlin-deficiency). Symptoms manifest in a muscle-specific manner, with the psoas and quadriceps muscles being some of the most affected by ∼8 months of age.

The most commonly studied dysf^−/−^ models are the naturally occurring A/J (A/J^dysf−/−^), SJL/J (SJL/J^dysf−/−^) and BLA/J (B6.A-Dysf^prmd^/GeneJ) mice. In addition, genetically modified knockout strains are also available; for example, the 129-Dysf^tm1Kcam^/J strain, which is also available in a C57BL/6J background (B6.129-Dysf^tm1Kcam^/J).

Both the A/J and SJL/J mice have impairments that are not observed in dysferlinopathy patients or other dysf^−/−^ mice (Ho et al., 2004). These include poor fertility and susceptibility to infection, which are proposed to be because of unknown modifiers within the genetic backgrounds rather than the dysferlin deficiency itself (Doetschman, 2009). Thus, the A/J and SJL/J dysf^−/−^ mice were backcrossed onto the better-defined genetic backgrounds, C57BL/6J (producing the BLA/J mouse) and C57BL/10J strains, respectively; also providing each new strain with a genetically defined dysferlin-positive WT control.

Earlier studies, many of which were conducted before these strains were recognized as dysferlin-deficient, used A/J and SJL/J mice (identified by 2004 and 1999, respectively; see Table 1). More recently, the BLA/J mouse has become the more popular model owing to its similar phenotype to other dysf^−/−^ models, reduced susceptibility to infections and the well-studied C57BL/6J background (Lostal et al., 2010).

Increased lipofuscin, a classical measure of cumulative oxidative damage, is an early histological change in dysf^−/−^ muscles, detected at 3 months in A/J mice (Terrill et al., 2013). Marked histopathology is evident in selected muscles (psoas>quadriceps) by 8 months in all dysf^−/−^ mice, with replacement of myofibres by adipocytes, which is more pronounced in older mice – studied up to 29 months of age (Albrecht et al., 2011; Hornsey et al., 2013; Terrill et al., 2013). However, the replacement of myofibres by adipocytes is not readily explained by myonecrosis, as this is relatively low (Terrill et al., 2013). The presence of conspicuous lipid droplets within dysf^−/−^ myofibres of rodents and humans is recognized as a striking feature (Demonbreun et al., 2014; Grounds et al., 2014). Recent lipidomic studies in young BLA/J mice showed marked changes in lipid metabolism and lipid composition of dysf^−/−^ muscles (Haynes et al., 2019).

Hornsey et al. provide a good review of the classic dysf^−/−^ mouse models (Hornsey et al., 2013), and details of many dysf^−/−^ strains are further provided on the Jain Foundation webpage (https://www.jain-foundation.org/scientific-resources/research-tools/mouse-models-dysferlin-deficiency).

Dysf^−/−^ mice have also been crossed with many other strains that lack specific genes to further understand the role of dysferlin and associated proteins in disease pathogenesis, for example the C3-deficient (Han et al., 2010), dystrophin-deficient *mdx* (Han et al., 2011), myoferlin-null (Demonbreun et al., 2014), annexin A2 knockout (Defour et al., 2017) and ApoE-null mice (Sellers et al., 2018).

## Sarcoglycanopathies

The α-, β-, γ- and δ-sarcoglycans are structural muscle proteins that are absent in sarcoglycanopathy patients, underlying progressive muscle wasting that manifests as LGMD R3, R4, R5 and R6, respectively (previously known as 2D, 2E, 2C and 2F). In sarcoglycanopathy patients, the medium age of onset is 6-8 years. The *Sgca-*null, *Sgcb-*null, *Sgcg-*null and *Sgcd-*null (Coral-Vazquez et al., 1999; Hack et al., 2000) mice are the classic models used to study the pathology of the α-, β-, γ- and δ-sarcoglycanopathies, respectively. These models display progressive muscle pathology and functional impairments of variable severity starting at 1 week of age, thereby emulating the human disease fairly accurately. Notably, all sarcoglycan-null models except the *Sgca*-null mice develop a cardiac phenotype from as early as 8 weeks (Coral-Vazquez et al., 1999; Durbeej et al., 2000; Hack et al., 1998).

As in the *mdx* strain, the pathology of *Sgcg-*null mice is more severe when crossed onto the DBA2/J genetic background (Heydemann et al., 2005). Recently, a novel variation of the *Sgcg-*null mouse has been generated, which, for the first time, allows investigation of exon skipping therapy for R5 γ-sarcoglycan-related LGMD patients (LGMD2C). The 521ΔT mouse has a single nucleotide deletion in exon 6, corresponding to the most common mutation found in patients (Demonbreun et al., 2019). Multi-skipping of exons 4, 5, 6 and 7 is required to restore the open reading frame, resulting in the expression of mini-γ sarcoglycan in these mice.

## Congenital muscular dystrophies (CMDs)

CMDs are a large group of muscular dystrophies with an early age of onset. Here, we focus on two common CMDs that manifest at birth. In humans, defects in the α2 chain of laminin (also called merosin) and α-dystroglycan underlie merosin-deficient congenital muscular dystrophy type 1A (MDC1A) and the dystroglycanopathies, respectively (Durbeej, 2015; Gawlik and Durbeej, 2011; Saunier et al., 2016).

For laminin α2-deficiency, five mouse models have been regularly studied, with three described in Table 1: two knockouts (*dy^3K^/dy^3K^* and *dy^w^/dy^w^*) (Kuang et al., 1998a; Miyagoe et al., 1997), two spontaneous models (*dy/dy* and *dy^2J^/dy^2J^*) (Meier and Southard, 1970; Michelson et al., 1955) and an ENU-induced model (*dy^7J^/dy^7J^*) (Patton et al., 2008). They have mild to moderate muscular dystrophy evident at birth, with peripheral neuropathy and severely impaired life expectancy (5-12 weeks for *dy^w^/dy^w^* and 3 weeks for *dy^3K^/dy^3K^* mice), except for the *dy^2J^/dy^2J^* and *dy^7J^/dy^7J^* strains (life span of >6 months). The *dy^w^/dy^w^* strain is most commonly used, and the TREAT-NMD consortium have generated several SOPs for this model (https://treat-nmd.org/research-overview/preclinical-research/sops-for-cmd-animal-models/).

Detailed information on dystroglycanopathy models is provided on the Cure-CMD webpage (https://www.curecmd.org/resources-for-scientists).

## Facioscapulohumeral muscular dystrophy (FSHD)

FSHD primarily affects the facial, shoulder and upper arm muscles. There is a large spread in the age of onset. Although most patients develop symptoms at ∼20 years of age, manifestations have been reported from infancy to 50 years of age. FSHD is caused by the epigenetic de-repression of the *DUX4* retrogene encoded within each unit of the D4Z4 macrosatellite repeat array. The complex underlying genetics (Daxinger et al., 2015) have prevented the generation of a single mouse model that would represent the genetic and pathologic aspects of the human disease. There are several mouse models available where each recapitulates only specific aspects of the disease (Lek et al., 2015). As the D4Z4 repeat array encoding the *DUX4* retrogene is specific to old-world primates, meaning that DUX4 is not expressed in mice (Leidenroth and Hewitt, 2010), this required the introduction of an exogenous genetic *DUX4* construct. The first FSHD mouse model, the D4Z4-2.5 mouse, carries a contracted pathogenic FSHD allele of two and a half copies of the D4Z4 repeat unit. DUX4 expression can be detected in both skeletal muscles and non-muscle tissues and the D4Z4 locus is hypomethylated, as in FSHD patients. However, the mice do not present muscle weakness or wasting, which may partly be explained by the very low DUX4 expression levels in their skeletal muscles (Krom et al., 2013). More recently, several DUX4-inducible mice have been generated, which consequently show a dose-dependent severity of muscle histopathology and functional impairments (Bosnakovski et al., 2017; Giesige et al., 2018; Jones and Jones, 2018). It has, however, become apparent that the activation of the downstream targets of *DUX4* in mice differs from that in humans. Finally, several xenograft models are also available, in which skeletal muscle tissue from FSHD patients (Chen et al., 2016; Zhang et al., 2014) or muscle precursor cells (Mueller et al., 2019; Sakellariou et al., 2016) are transplanted into a muscle of the mouse.

## Oculopharyngeal muscular dystrophy (OPMD)

OPMD is a late-onset monogenic myopathy primarily affecting the eyelid and pharyngeal muscle groups, with symptom manifestation from 40-60 years of age. The genetic cause of the disease is an expansion of the alanine track at the N-terminus end of the gene encoding for poly(A) binding protein nuclear 1 (*PABPN1*). OPMD has been identified throughout the world. Most reported cases are autosomal dominant, but several recessive cases have also been reported (Brais, 2009; de Leeuw et al., 2019). On the protein level, the alanine expansion varies between +1 and +8 over the non-pathogenic 10 alanine track. The expanded PABPN1 forms insoluble nuclear aggregates, which represent the histopathological hallmark of the disease (Trollet et al., 1993). The first mouse models for OPMD were generated with a high and constitutive overexpression of the 17 alanine-expanded PABPN1, of which the A17.1 mouse is the most well-studied model (Davies et al., 2010). Studies in this mouse and in cellular models that were generated with overexpression of the expanded PABPN1 showed induction of cell death (Davies et al., 2006, 2010). However, cell death is not observed in the muscles of OPMD patients. Moreover, unlike the age-associated disease progression in OPMD patients, progression of muscle pathology in the A17.1 mouse is attenuated with age (Trollet et al., 2010). Interventions aimed at reducing PABPN1 aggregation were beneficial in mouse models that were generated by high overexpression. Whether these interventions are also beneficial for OPMD patients remains unresolved. Recently, Vest et al. generated a knock-in mouse model of Ala17, which captures some of OPMD pathological hallmarks (Vest et al., 2017). Additional studies in these mice are required to assess whether this is a good model for OPMD. So far, it is unclear whether PABPN1 aggregates are toxic and directly cause muscle weakness in OPMD. Several studies demonstrated that when PABPN1 expression levels are significantly reduced, below a certain threshold, it leads to muscle atrophy and wasting (Olie et al., 2019; Riaz et al., 2016; Vest et al., 2017). In muscles of OPMD patients, levels of PABPN1 correlate with disease progression (Anvar et al., 2013). It has been suggested that muscle weakness in OPMD is caused by a combination of accumulation and aggregation of expanded PABPN1 and an age-associated reduction in PABPN1 expression levels, which together reduce the availability of normal PABPN1 below a functional threshold (Raz and Raz, 2014). However, thus far, there is no animal model that emulates this combinatorial condition as in OPMD patients.

## Conclusions

The availability of a variety of MD mouse models has greatly improved our understanding of pathogenesis and enabled the (pre)clinical development of several therapeutic approaches. Although these models allowed unprecedented opportunities for fundamental and applied research, their ever-increasing number also adds to the complexity of selecting the most appropriate model for a particular research question. The suitability of a certain model not only depends on the existence of the same genetic defect, but also on how well it emulates specific aspects of the human disease. Unfortunately, many MD mouse models are limited in their presentation of the human pathologies. These limitations, therefore, also add to the fact that the effects of a drug observed in mice may not necessarily predict the outcome in the clinical setting. To partly overcome these issues, the availability of natural life-history data for mouse models and of standardized operational procedures for *in vivo* outcome measures are pivotal for accurate study design and execution of high-quality preclinical research. Fortunately, these issues have received more attention in the last decade and are now in place for some MDs.

This article is part of a special subject collection ‘A Guide to Using Neuromuscular Disease Models for Basic and Preclinical Studies’, which was launched in a dedicated issue guest edited by Annemieke Aartsma-Rus, Maaike van Putten and James Dowling. See related articles in this collection at http://dmm.biologists.org/collection/neuromuscular.
